# Applications, ethics, and future agenda of generative artificial intelligence (GenAI) in tourism and hospitality: a systematic review

**DOI:** 10.3389/frai.2026.1935676

**Published:** 2026-10-29

**Authors:** Wai In Ho, Yifen Yin, Yuanyuan Yu, Haoqian Hu, Jingwen Cai, Chunning Wang

**Affiliations:** Faculty of Humanities and Social Sciences, Macao Polytechnic University, Macau, Macao SAR, China

**Keywords:** algorithm aversion, ethical risks, generative artificial intelligence, human-in-the-loop, systematic literature review, tourism and hospitality

## Abstract

Generative artificial intelligence (GenAI) is reshaping how tourism information is produced, exchanged, and consumed, yet its application scenarios, ethical consequences, and implications for service authenticity remain dispersed across a fast-growing literature. Following the PRISMA 2020 protocol, this study identifies 3,825 records from the Web of Science Core Collection and Scopus and conducts a thematic synthesis of 461 publications, addressing three questions: where GenAI creates value on the supply and demand sides of tourism and hospitality, which ethical risks and organizational dilemmas accompany its adoption, and how machine intervention conflicts with interactional authenticity in service encounters. The synthesis indicates that GenAI operates as a double-edged sword. On the demand side, conversational and multimodal systems lower information search and decision-making costs; on the supply side, automated content generation unlocks operational capacity—yet these gains coexist with algorithmic bias and hallucination, privacy trade-offs, and employee deskilling. In service interactions, the computational essence of machine-generated empathy can elicit expectancy violations and algorithm aversion, weakening the interpersonal authenticity on which hospitality depends. The review integrates these findings into an efficacy-risk hedging framework embedded in a socio-technical account of human-AI collaboration and derives a tiered human-in-the-loop governance model. Its primary contribution is to link application-level benefits, systemic risks, and the authenticity conflict within one evidence-based framework, with each claim anchored in article-level evidence counts.

## 1 Introduction

Since the emergence of generative artificial intelligence (GenAI) models in late 2022, technological advancements spearheaded by these tools have transformed the operational paradigms of the information industry. Distinct from traditional algorithms that primarily rely on historical data for probabilistic forecasting, GenAI possesses capacities for semantic comprehension and multimodal generation (Carvalho and Ivanov, 2024). Enhanced computational power has propelled AI systems from mere auxiliary tools to direct participants in content production and conversational interaction (Sigala et al., 2024). As these systems acquire the capability to execute creative tasks independently, the transmission of information and the distribution of value within tourism services have consequently shifted toward an algorithm-driven paradigm (Bilgihan et al., 2024).

Because tourism services are quintessential experiential goods, the delivery of their core value relies heavily on interpersonal interaction and the circulation of authentic information (Carvalho and Ivanov, 2024). The deployment of GenAI disrupts the pre-existing equilibrium between supply and demand, altering industry operational mechanisms. On the tourist side, multimodal dialogue systems convert unidirectional keyword searches into customized interactions, reshaping information retrieval pathways (Volchek and Ivanov, 2024). Simultaneously, to navigate the instability of labor supply, enterprises incorporate automation technologies to process customer inquiries and generate marketing content, thereby maintaining the stability of service workflows (Luo et al., 2025). AI-driven synthetic service experiences are progressively penetrating human-dominated service spaces, blurring the boundaries between technology and service behavior (Dwivedi et al., 2024).

Accompanying the widespread application of GenAI technologies, empirical research proliferated between 2023 and 2026, and several reviews have begun to map this territory. Earlier contributions differ from the present review in scope and method: some are expert agenda pieces rather than systematic syntheses (Dogru et al., 2025; Sigala et al., 2024; Dwivedi et al., 2024), others focus on ChatGPT or conversational AI specifically (Tuyen et al., 2025; Aransyah et al., 2026), and integrative reviews either stop at 2024 evidence or emphasize bibliometric mapping over thematic synthesis (Li et al., 2025; Malekpour et al., 2026). What remains missing is a review that covers the full class of generative technologies across the entire tourist journey, links application-level benefits to systemic risks in one analytical framework, and grounds every thematic claim in article-level evidence counts. Current inquiries also predominantly treat GenAI as an exogenous variable, largely adopting established technology acceptance models to measure user adoption intentions (Aransyah et al., 2026), or concentrating on the risks of bias and hallucination in algorithmic recommendations (Ren et al., 2025; Rejón-Guardia et al., 2026). Lacking a comprehensive analytical framework, the existing literature struggles to explain the conflicts between technological efficiency and ethical norms. The precision of customized services often clashes with users’ psychological need for privacy protection (Chakraborty et al., 2025), while the job insecurity and risk of skill obsolescence experienced by practitioners facing GenAI intervention remain systematically unaddressed (Lu et al., 2024; Zhao et al., 2024). Moreover, the use of synthetic content tends to dilute the authenticity emphasized by the tourism industry (Ali et al., 2025), subsequently triggering algorithm aversion and trust erosion among consumers (Tan et al., 2025). Sustaining the emotional value of services within an automated environment constitutes a critical bottleneck that contemporary tourism and hospitality management research urgently needs to overcome (Loureiro et al., 2025).

Addressing these theoretical gaps, this study systematically reviews and synthesizes the empirical evidence. The primary contribution of this review is an integrated efficacy-risk hedging framework: it links application-level benefits, systemic ethical risks, and the authenticity conflict into a single socio-technical account, with each component anchored in article-level evidence counts. To construct a rational human-AI collaboration model while enhancing operational efficiency—and to clarify future evolutionary directions—this review addresses three core questions: First, across both the supply and demand sides, in which specific scenarios is GenAI predominantly applied within the tourism and hospitality industry, and how is its application value realized? Second, what ethical risks (e.g., cognitive biases and privacy trade-offs) and organizational management dilemmas does the application of this technology raise? Third, within human-centric tourism services, how does technological intervention conflict with interactional authenticity? Methodological limitations of the reviewed literature and the future research agenda are developed separately in the subsequent discussion.

## 2 Literature review

Investigating the interplay between technological applications and ethical challenges necessitates a systematic review of existing empirical evidence. Constructing a socio-technical evaluation model that integrates multi-dimensional perspectives depends on the systematic extraction and reorganization of this evidence. This section delineates the literature acquisition and screening procedures. By analyzing metadata characteristics, it characterizes the field’s evolutionary trajectory, thereby establishing an empirical foundation for subsequent thematic discussions.

### 2.1 GenAI adoption and value creation in tourism and hospitality

Since the public release of generative models in late 2022, research on their tourism applications has expanded from exploratory commentary to a large empirical base. The demand-side literature examines how conversational and multimodal systems reshape information search and itinerary planning. Early studies documented the shift from keyword-based search to natural-language dialogue and showed that perceived usefulness, trust, and ease of use drive adoption (Wong et al., 2023; Kim et al., 2024), while later work increasingly stresses the contingency of acceptance: tourists use these systems for inspiration and comparison, but they scrutinize recommendations when decisions involve bookings or risk (Kim et al., 2025; Bingöl, 2026). Recent contributions extend this line with evaluations of the factual quality of AI-generated tourism information (Dong et al., 2026), evidence on switching behavior from online travel communities to generative services (Xiao et al., 2026), and typologies of human-GenAI interaction patterns (Zhao et al., 2026). On the supply side, research examines automated review analysis, marketing content production, guest-service responses, and back-office functions (He et al., 2026; Shen et al., 2026a; Zhang et al., 2024), documenting efficiency gains while noting that AI-generated content can differ from professional content in ways that matter for brand communication.

### 2.2 Ethics and GenAI in tourism and hospitality

A second stream addresses the ethical consequences of generative systems. Studies document how hallucinated or outdated information undermines trust and distorts decisions (Christensen et al., 2025; Kim et al., 2025), how popularity bias concentrates demand in already-crowded destinations (Mellors, 2026; Paül i Agustí, 2025), and how cultural and identity biases enter generated destination imagery (Zhu et al., 2025; Manor and Segev, 2025). Privacy research shows that personalization depends on increasingly fine-grained personal data and that privacy concerns amplify threat perceptions and usage avoidance (Morosan, 2026; Yhee et al., 2025), while recent work configures trust and distrust as distinct mechanisms rather than opposites (Jeong et al., 2026). The organizational stream documents job insecurity, techno-overload, skill erosion, and accountability ambiguity among employees (Lu et al., 2024; Zhao et al., 2024; Selem et al., 2027; Wei et al., 2026), and a growing governance literature translates these risks into responsible-AI requirements (Kwok et al., 2025; Huang et al., 2026). Subsequent analysis organizes these dimensions into an explicit three-tier classification with evidence counts, so that synthesized dimensions are distinguished from those that remain under-researched in tourism.

### 2.3 Authenticity and human-AI interaction

A third stream examines what happens when machine-generated communication replaces human emotional labor. Parasocial interaction research shows that anthropomorphic cues can generate emotional bonds with conversational agents (Duong et al., 2025; Loureiro et al., 2025), but disclosure of machine authorship and detection of scripted empathy can trigger expectancy violations and algorithm aversion (Tan et al., 2025; Aransyah et al., 2026). The authenticity literature distinguishes objective, constructive, existential, brand, and interactional authenticity (Wang, 1999); in algorithmic service contexts it is interactional and brand authenticity that are most exposed, because the perceived genuineness of the exchange—not the toured object—is at stake. This stream motivates the human-in-the-loop question that runs through the present review: which interactions can be delegated to machines without eroding the human-centric basis of hospitality.

### 2.4 Prior reviews and the positioning of this study

Several recent reviews map adjacent territory. Expert agenda pieces outline opportunities and challenges without systematic searches (Dogru et al., 2025; Sigala et al., 2024; Dwivedi et al., 2024). Systematic efforts either focus on ChatGPT or conversational AI specifically (Tuyen et al., 2025; Aransyah et al., 2026) or emphasize bibliometric mapping over thematic synthesis (Li et al., 2025; Malekpour et al., 2026). Relative to these reviews, the present study differs along every dimension of comparison: it covers a longer publication window (2021 through September 9, 2026), searches two comprehensive databases (the Web of Science Core Collection and Scopus) under the PRISMA protocol, and synthesizes a substantially larger sample of 461 publications; in contrast to the expert agenda pieces, its thematic claims are grounded in systematic screening, quality appraisal, and article-level evidence counts. Three gaps follow. First, no review covers the full class of generative technologies—text, image, and multimodal—across the entire tourist journey. Second, reviews that map the field rarely link application-level benefits to systemic risks within one analytical framework. Third, thematic claims are seldom anchored in article-level evidence counts and quality appraisal. The three research questions specified in the Introduction are designed to close these gaps.

### 2.5 An integrative socio-technical framework

The empirical findings synthesized throughout this review can be organized within a single socio-technical framework that spans actors, levels, and mechanisms. The actors are tourists, frontline employees, firms and destination management organizations, destinations and local communities, and regulators. The analysis operates at four levels: the individual level (psychological mechanisms), the interactional level (service encounters), the organizational level (work design and governance), and the institutional level (regulation and industry structure). At the core of the framework sits a technological layer defined by GenAI affordances and constraints: the affordances of semantic comprehension, multimodal generation, and personalization enable low-cost, context-aware content production; the constraints of hallucination proneness, opacity, and the absence of spatial awareness bound what these systems can deliver.

A psychological-mechanism layer mediates between the technological layer and observable outcomes. The adoption theories that dominate the literature—TAM and UTAUT—capture baseline evaluations of usefulness and ease of use on the demand side. Conservation of Resources theory and Technology Threat Avoidance Theory extend this logic to threat appraisal: employees weigh technological assistance against substitution risk, and tourists weigh personalization against privacy loss. At the interactional level, Parasocial Interaction theory explains how anthropomorphic cues generate emotional bonds, Expectancy Violation theory explains why the disclosure of computational origins breaks those bonds, and the embodied-cognition perspective explains why emotionally complex encounters exceed the capacities of disembodied models. These theories are therefore not rival explanations but complementary mechanisms operating at different layers of the same socio-technical system: adoption theories at the utility layer, resource and threat theories at the appraisal layer, and interaction theories at the emotional layer.

An organizational-process layer translates these mechanisms into design choices: task allocation between humans and AI, disclosure policies, privacy controls, training, and audit arrangements. Outcomes then emerge as the joint product of all layers: efficiency gains and decision-cost reductions on one side, and trust erosion, job insecurity, skill obsolescence, authenticity loss, and spatial or cultural inequities on the other. Boundary conditions moderate the entire system: task involvement, disclosure, digital literacy, culture, firm size, and regulatory context determine which outcomes dominate in a given setting.

## 3 Literature screening and metadata analysis

### 3.1 Literature search and screening process based on the PRISMA statement

This study employed the Preferred Reporting Items for Systematic Reviews and Meta-Analyses (PRISMA 2020) framework to ensure the transparency of the screening process and the reproducibility of the findings. Because generative AI research diffuses rapidly across disciplines, literature was retrieved from two multidisciplinary databases: the Web of Science Core Collection (covering the Science Citation Index Expanded, the Social Sciences Citation Index, the Emerging Sources Citation Index, the Arts and Humanities Citation Index, and the Conference Proceedings Citation Indexes) and Scopus. The search was conducted in two rounds. The initial round, with a cutoff date of March 22, 2026, used the following query in the Web of Science Core Collection: TS = (“generative AI” OR “generative artificial intelligence” OR “GenAI” OR “ChatGPT” OR “large language model*” OR “LLM*”) AND TS = (“tourism” OR “tourist*” OR “hospitality” OR “hotel*” OR “travel*” OR “destination management”). A supplementary round was performed on September 9, 2026 in response to the risk that the initial keyword set could omit studies using related technological terminology. The supplementary queries added foundation models, GPT-3, GPT-4, GPT-5, generative agents, diffusion models, text-to-image systems, and AI-generated content, and were run in both databases: in the Web of Science Core Collection, TS = (“generative AI” OR “generative artificial intelligence” OR “GenAI” OR “ChatGPT” OR “large language model*” OR “LLM*” OR “foundation model*” OR “GPT-3” OR “GPT-4” OR “GPT-5” OR “generative agent*” OR “diffusion model*” OR “text-to-image” OR “AI-generated content” OR “AIGC”) AND TS = (“tourism” OR “tourist*” OR “hospitality” OR “hotel*” OR “travel*” OR “destination management”) AND PY = 2021–2026; in Scopus, the corresponding query was TITLE-ABS-KEY(“generative AI” OR “generative artificial intelligence” OR “GenAI” OR “ChatGPT” OR “large language model*” OR “LLM*” OR “foundation model*” OR “GPT-3” OR “GPT-4” OR “GPT-5” OR “generative agent*” OR “diffusion model*” OR “text-to-image” OR “AI-generated content” OR “AIGC”) AND TITLE-ABS-KEY(tourism OR tourist* OR hospitality OR hotel* OR travel* OR “destination management”) AND PUBYEAR > 2020 AND PUBYEAR < 2027. Both rounds covered records published from January 1, 2021 to the supplementary search date. No language or document-type restrictions were imposed at the search stage: journal articles (including early-access records), conference papers, and book chapters were all retrieved and evaluated against the eligibility criteria, and early-access articles were treated as eligible publications. Duplicate records across the two databases were removed by DOI and, when DOIs were absent, by normalized title. Backward and forward citation searching was not performed; this is acknowledged in the limitations.

The initial round retrieved 742 records. The supplementary round retrieved 1,500 records from the Web of Science Core Collection and 2,325 records from Scopus (3,825 records in total); after removing 1,283 duplicates across the two databases, 2,542 unique records remained, of which 765 had already been assessed in the initial round. The remaining 1,777 records entered the screening process described below. To extract a core sample relevant to tourism management and ethical dimensions, the research team applied a hierarchical exclusion procedure comprising five exclusion categories. Each category was defined by an explicit decision rule, so that records deviating from the managerial perspective or the technological essence of the review were filtered out consistently:

1. Invalid document type. Records that are not original academic publications—meeting abstracts, book reviews, editorials, front matter, or records without abstract information—were excluded. For example, a conference abstract for an LLM-based orienteering system that contained no abstract text was excluded under this category. Journal articles, conference papers, and book chapters reporting original research or systematic synthesis were eligible, including early-access versions of journal articles.
2. Out of context. Records whose research subject and application scenario fall outside tourism, hospitality, conventions, or destination management were excluded even when they examined generative models. For example, studies of universal human-mobility prediction using foundation models and studies of reaction–diffusion equations in mathematical biology were excluded. Tourism applications were defined as traveler-facing and destination-level uses; hospitality applications encompassed accommodation, food service, and tourism-related service delivery. Applications in transport, retail, or healthcare were excluded unless the tourism or hospitality context was the research setting.
3. Out of intervention. Records focusing on traditional predictive AI without substantive engagement with generative capabilities were excluded. Generative AI was operationalized as models that produce new content (text, images, or other modalities) from learned distributions, including large language models, text-to-image systems, and diffusion models. Predictive analytics, regression- or machine-learning-based demand forecasting, and recommendation algorithms without generative components were excluded unless they evaluated a generative system. Large language models were distinguished from conventional conversational AI: rule-based or retrieval-based chatbots were out of scope, whereas LLM-based conversational systems were in scope. For example, an econometric market forecast of the Airbnb sharing economy was excluded, whereas a study of ChatGPT-based itinerary generation was included.
4. Lack of disciplinary perspective. Records confined to computer science or engineering objectives—parameter optimization, algorithm derivation, or benchmark testing—were excluded unless they addressed human-computer interaction, service recovery, or ethical dimensions such as hallucination risks or data privacy. For example, a benchmark evaluation of geo-spatial reasoning in foundation models without any user- or service-level analysis was excluded; by contrast, an LLM-driven aspect-based sentiment analysis of hotel service failures was included because it addressed service management outcomes.
5. Out of focus. Records that satisfied the preceding categories but mentioned generative technology only in passing within the background section were excluded, because the actual research focus diverged from the review’s themes. For example, a study of how cultural identity shaped tourism intention, in which generative AI appeared only in the background discussion, was excluded.

The literature screening followed a two-stage independent duplicate screening procedure. In the initial round, two researchers independently screened the titles and abstracts of the 742 records; the raw agreement rate was 95.69% with a Cohen’s kappa of 0.91, and disagreements were resolved through discussion with a third researcher. This round excluded 362 records: 246 for contextual deviation, together with invalid document types, studies of traditional predictive AI, STEM-oriented studies lacking a managerial perspective, and records in which generative technology was peripheral to the research focus. In the supplementary round, the same two researchers independently screened the titles and abstracts of the 1,777 newly identified records against the same criteria; the agreement rate was 94.1% with a Cohen’s kappa of 0.80, and disagreements were again resolved through discussion with a third researcher. The supplementary screening excluded 1,544 records: 233 invalid document types, 845 out of context, 135 studies of traditional predictive AI, 262 lacking a disciplinary perspective, and 69 out of focus, and retained 233 records.

In the second stage, the full texts of the retained records were assessed against the same eligibility criteria. In the initial round this stage excluded 152 articles, which consisted mainly of traditional tourist-flow forecasting or dynamic-pricing studies without multimodal interaction features and algorithm-derivation papers lacking a management science perspective, leaving 228 eligible publications; the agreement rate was 91.84% with a Cohen’s kappa of 0.83. In the supplementary round, the full texts of the 233 retained records were assessed in the same way, and all 233 were confirmed eligible. Combining both rounds, 461 publications were ultimately included for thematic synthesis and theoretical construction (see Figure 1).

**Figure 1:**
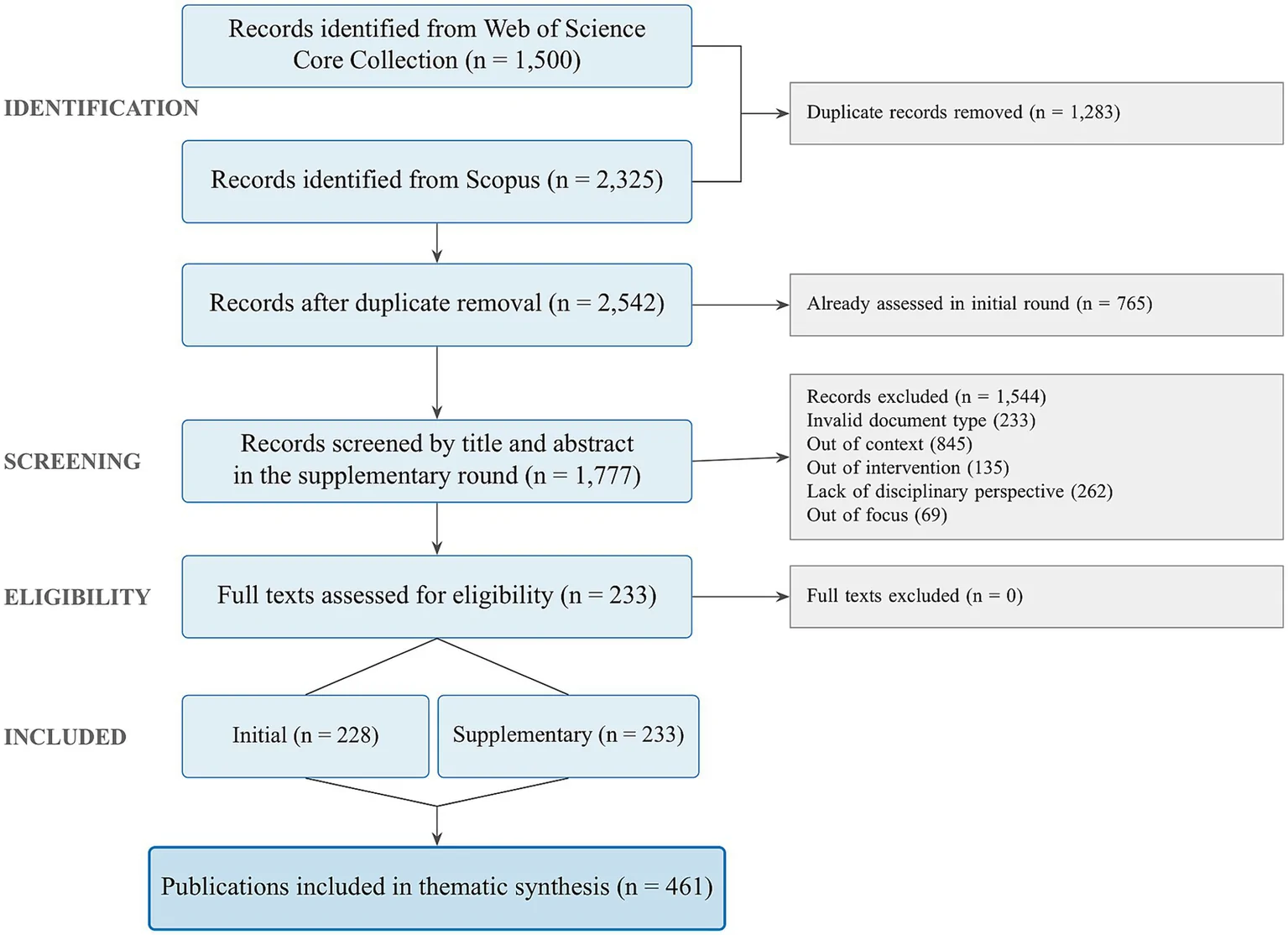
PRISMA 2020 flowchart of the literature screening process (initial and supplementary rounds combined).

### 3.2 Data extraction and thematic coding

A structured extraction form was developed and implemented in Microsoft Excel and Python (pandas). For each included publication, the coders recorded seven fields: (1) study region, coded by country (full names, with multi-country comparisons separated by slashes) or as Global or Theoretical; (2) research method, coded into one of four mutually exclusive categories—quantitative, qualitative, mixed methods, or theoretical/review; (3) the tourist journey stage examined (pre-trip, on-trip, post-trip, or whole-journey); (4) application scenarios and value creation of GenAI; (5) ethical risks and management dilemmas; (6) reflections on authenticity and human touch; and (7) the research agenda proposed by the authors. Fields (4)–(7) were coded as concise summaries of the publication’s substantive findings rather than free commentary.

The coding procedure combined deductive and inductive moves. The three research questions supplied the initial higher-order categories; application scenarios, ethical dimensions, and authenticity-related themes were then refined inductively through iterative reading of the coded summaries. Two coders were trained on the coding protocol and completed a pilot round on 20 records to align their interpretations. Coding disagreements were resolved through discussion, and a third coder arbitrated where consensus was not reached. To assess the reliability of the thematic coding, both coders independently coded a random subsample of 50 records: agreement on research method was 92.0% (Cohen’s kappa = 0.87), agreement on journey stage was 80.0% (kappa = 0.69), and agreement on section assignment was 60.0% for exact matches, with at least one overlapping section in 100% of the subsample; remaining disagreements were resolved by consensus. Contradictory findings and negative cases were not discarded: they were retained in the extraction matrix and are reported wherever they qualify the dominant pattern. The complete coding dictionary, the article-level extraction matrix, and all screening decisions are available in the supplementary repository.

### 3.3 Quality appraisal

To avoid treating all evidence as equally credible, a design-sensitive quality appraisal was conducted following the logic of the Mixed Methods Appraisal Tool. Four design-specific checklists were applied: for quantitative studies, sampling and sample size, measurement quality, and the match between the analysis and the research question; for qualitative studies, the adequacy of data-collection and analysis procedures and the presence of rigor measures such as coder reliability or member checking; for mixed-method studies, the rationale for integrating the quantitative and qualitative components; and for theoretical or review publications, the systematicity of the search and the clarity of the theoretical argument. Two reviewers independently rated each of the 461 publications as high, moderate, or low on the basis of the methodological information documented in the standardized data extraction form, and disagreements were resolved by consensus. Publications whose documented information indicated neither particular methodological strengths nor evident weaknesses were rated moderate. The distribution was 12 high (2.6%), 395 moderate (85.7%), and 54 low (11.7%). Interpretive weight in the synthesis was given primarily to high- and moderate-quality evidence.

### 3.4 Descriptive statistical analysis of core literature

Grounded in the PRISMA screening process, the 461 publications ultimately included constitute the foundational literature corpus for subsequent thematic discussions. Of these, 398 publications report empirical research (270 quantitative, 77 mixed-method, and 51 qualitative studies), and 63 are theoretical or review publications; theoretical and review publications were eligible because they map conceptual developments and research agendas for this emerging field, and they are reported separately from empirical findings in every analysis below. The unit of analysis throughout this review is the publication. Transcending the micro-level perspective of isolated studies, mapping the metadata distribution across publication years, research methodologies, and industry application scenarios characterizes the evolutionary trajectory of contemporary research priorities.

An examination of the temporal distribution of recent literature indicates that the proliferation of empirical research did not occur synchronously with the advent of GenAI models in late 2022. Instead, it exhibits a non-linear growth trajectory characterized by a distinct time-lag effect (see Figure 2).

**Figure 2:**
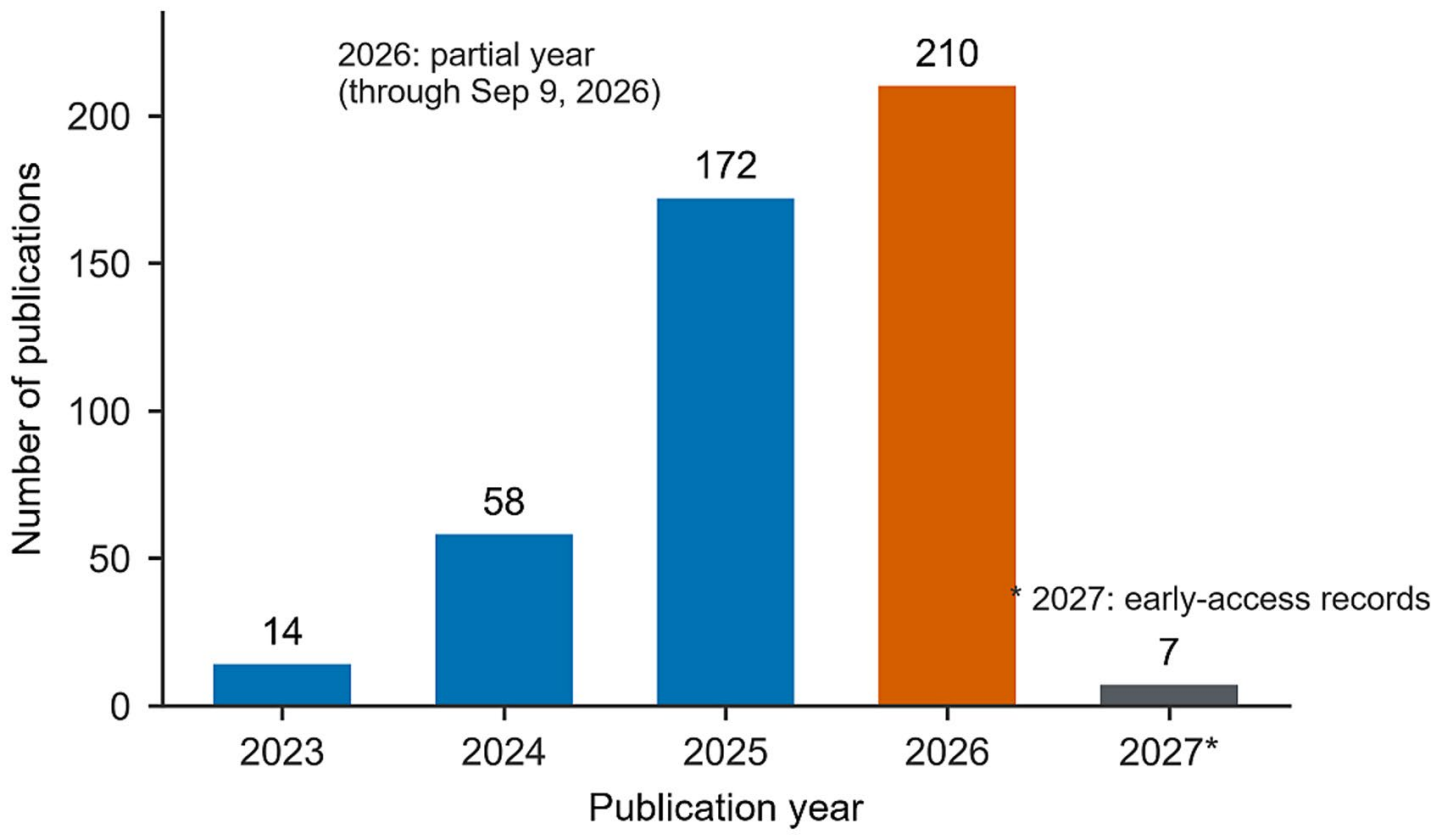
Publication year distribution of the 461 included publications. The 2026 bar covers a partial year (through September 9, 2026); 2027 denotes early-access records. No trend line is drawn because 2026 is incomplete.

As a sensitivity check, all descriptive analyses were recomputed after excluding the 54 low-quality publications. The number of studies supporting each thematic section retained between 86.0 and 90.4% of its full-sample value, and the distributions of research methods, journey stages, and publication years remained materially unchanged. The main conclusions therefore do not depend on methodologically weak studies.

Publication volumes remained relatively low in 2023 (14 publications) and 2024 (58 publications). During this nascent phase, academia predominantly focused on prospective deductions of technological potential or preliminary explorations of ethical boundaries; the inherent duration of empirical research design and peer review cycles structurally delayed the publication of early findings (Buitrago-Esquinas et al., 2024; Sigala et al., 2024). Publication output then rose sharply to 172 publications in 2025 and 210 by the supplementary search date in 2026 (with 7 early-access records dated 2027), and the 2026 figure covers only a partial year. Two developments are consistent with this surge, although publication counts alone cannot confirm a causal role: the architectural expansion of GenAI from unimodal text generation toward multimodal interactive capabilities encompassing image and voice (Huang et al., 2024), and the commercial democratization of Application Programming Interfaces, which granted scholars access to real-world enterprise deployment cases and user interaction data (Carvalho and Ivanov, 2024). The discourse surrounding GenAI applications in tourism has moved beyond preliminary conceptual exploration and is establishing itself as a chronic managerial imperative concerning human-AI collaboration and service innovation. This maturation provides a robust empirical foundation for comprehensively assessing the technology’s commercial value and multidimensional challenges (Aransyah et al., 2026).

Despite this substantial expansion in research volume, methodological selection remains highly homogenized and oversimplified. Scrutinizing this structural imbalance helps identify the epistemological limitations embedded in current academic conclusions (see Figure 3).

**Figure 3:**
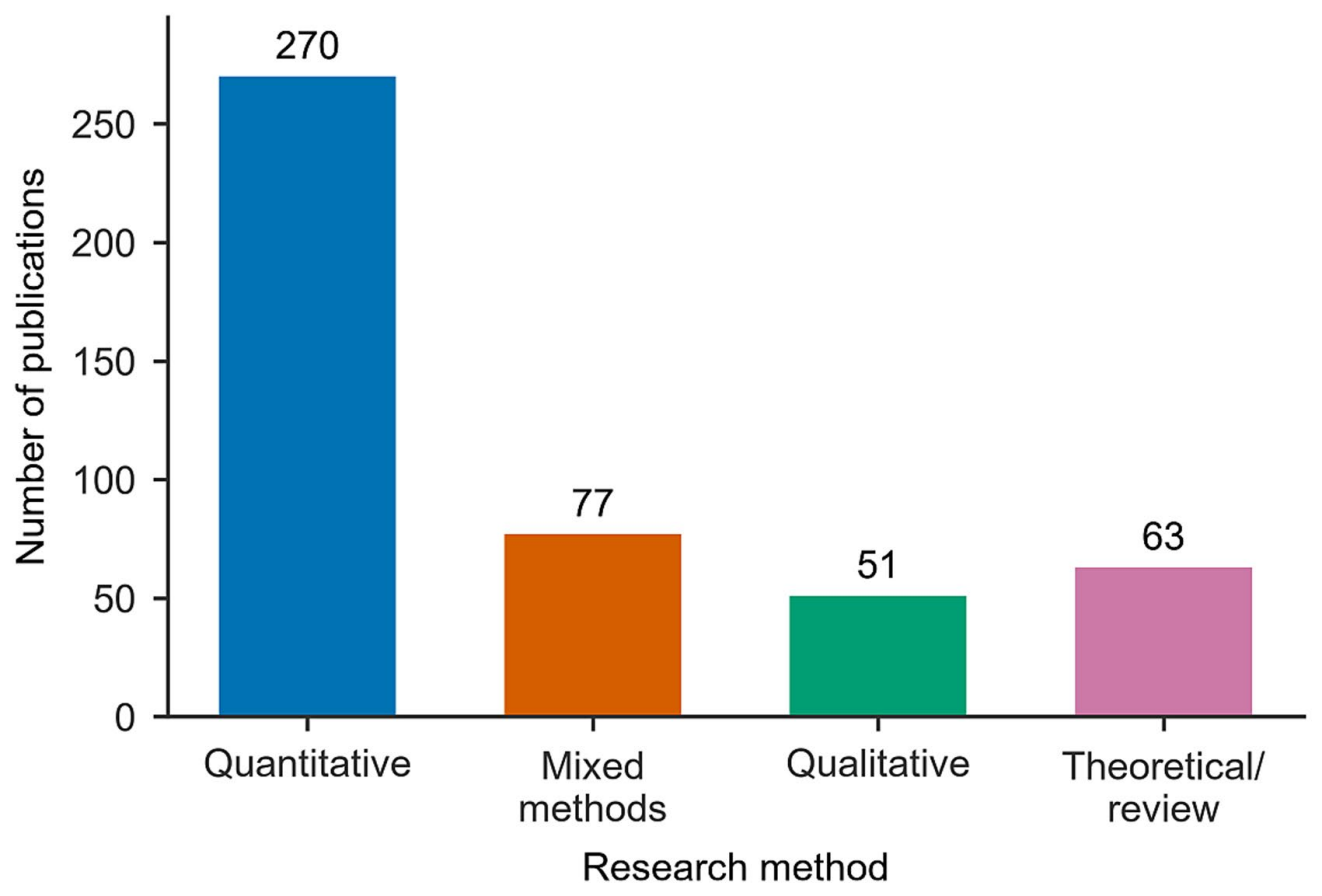
Distribution of research methods among the 461 included publications. Categories are mutually exclusive; mixed methods refers to designs integrating quantitative and qualitative components.

Quantitative analysis (270 publications) and mixed methods (77 publications) constitute the dominant trajectories of current research. A majority of the literature habitually reduces GenAI to a static independent variable. By adopting the Technology Acceptance Model (TAM) or the Unified Theory of Acceptance and Use of Technology (UTAUT), researchers frequently utilize structural equation modeling to batch-test consumers’ perceived usefulness or adoption intentions (Bilgihan et al., 2024; Ghorbanzadeh et al., 2025). While this highly standardized quantitative paradigm has rapidly accumulated an empirical baseline for the field, it reduces highly dynamic natural language interactions (e.g., prompt engineering, multi-turn conversational repair) to static measurement items, thereby neglecting the complex psychological negotiations inherent in human-AI collaboration. In contrast, purely qualitative studies (51 publications) and theoretical reviews (63 publications) remain marginalized. When confronting deep-seated techno-ethical dilemmas—such as algorithm aversion and privacy defensiveness triggered by machine-generated content—singular scale measurements struggle to capture the emotional tension and cognitive dissonance occurring within service scenarios. A lack of in-depth field observations, discourse analysis, and grounded theory interventions means the current literature lacks the theoretical penetration at the micro-mechanism level required to explain practitioners’ hidden anxieties and organizational adaptability crises in the face of technological substitution. This methodological imbalance underscores an urgent need for academia to transcend singular efficiency metrics and pivot toward more complex perspectives encompassing management science and technological ethics. The four methodological categories are mutually exclusive and exhaustive. Mixed-method publications were defined as those integrating quantitative and qualitative components within a single research design; publications reporting several separate studies were classified according to the design of their primary study.

Projecting these technological applications and methodologies onto specific stages of the tourism service journey shows pronounced spatiotemporal asymmetries in the literature distribution, further mirroring the deep-seated conflicts arising when technology intervenes in physical services (see Figure 4).

**Figure 4:**
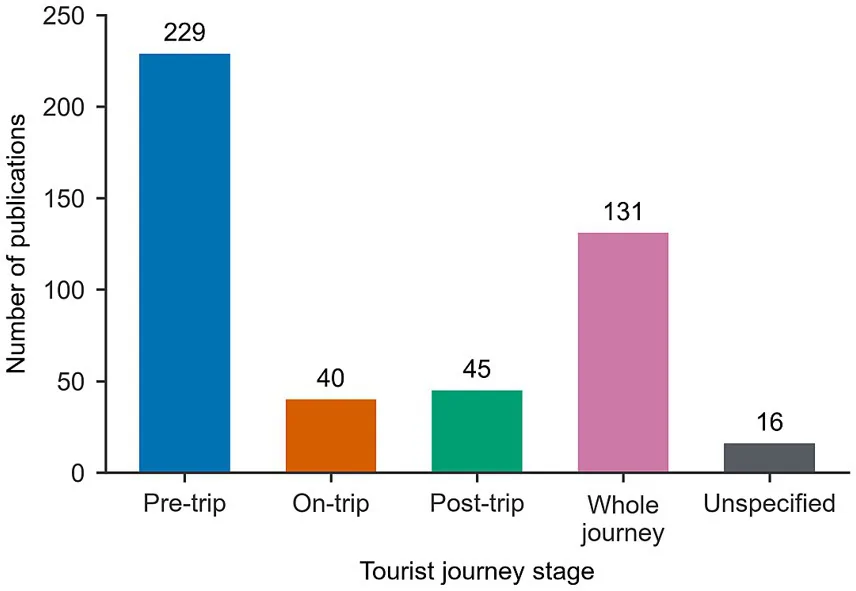
Distribution of the tourist journey stages examined in the 461 included publications.

Research focusing on the pre-trip stage (229 publications) is highly saturated, accounting for about half of the total sample. The exceptional semantic parsing and information aggregation capabilities of GenAI models align with the text-heavy information decision-making demands of the pre-trip phase, establishing it as the most observable and measurable application context (Luo et al., 2025). As tourists transition into the on-trip stage—which intrinsically involves physical destinations—the number of relevant studies remains comparatively small (40 publications). One plausible explanation is that pure GenAI systems lack spatial awareness of physical environments and real-time contextual comprehension: without integration with the Internet of Things (IoT) or embodied AI, GenAI models may struggle with the complex and uncertain physical interaction demands of the on-trip phase; however, this interpretation remains inferential, as the pattern could equally reflect measurement difficulty rather than a technological ceiling (Paül i Agustí, 2025). Literature focusing on the post-trip stage (45 publications) similarly accounts for a smaller proportion. Post-trip behavior typically entails the reconstruction of personal emotional memories and the cultivation of online reputation. AI-authored travelogues or reviews challenge the premises of objective authenticity and emotional sincerity within the tourism experience (Li and Bang, 2026). The crisis of content authenticity and the moral ambiguity triggered by this technological delegation exacerbate both the ethical risks and the measurement difficulties inherent in empirical research at this stage.

Beyond these distinct stages, the contextual settings of the remaining literature further expose an imprecise research focus. A total of 131 publications broadly generalize GenAI application across the entire tourist lifecycle. Such studies primarily rely on macro-strategic frameworks or generic variable models, failing to meticulously unpack the differentiated experiences occurring at specific service touchpoints. An additional 16 publications completely omit the specification of any application stage. Their discourse predominantly leans toward macro-level industrial impacts, policy regulations, or the construction of holistic technological frameworks, adopting a top-down, abstract perspective. Collectively, these distributional characteristics confirm that the scope of current research remains largely confined to information interactions on digital devices, yet to substantively permeate the core physical spaces that define the tourism industry as a tangible experience economy.

## 4 Application scenarios and value of generative artificial intelligence

While macroscopic literature statistics outline the external contours of technological evolution, a substantive evaluation of GenAI efficacy requires shifting the analytical lens to specific industrial application contexts. The underlying operational logic of the tourism industry relies on information transmission and supply–demand matching—a foundation of information exchange currently being reconstructed by the deployment of GenAI models. Within this context, technological intervention gives rise to a bidirectional structural transformation in information acquisition and content production (see Figure 5). On the demand side, tourists’ information retrieval gradually transitions from localized keyword matching to natural language dialogue (Mladenović et al., 2026a); on the supply side, corporate content manufacturing models concurrently migrate toward automated algorithmic generation (Almeida and Ivanov, 2024). To explore this dual transformation, the application evidence base across the following two subsections comprises 427 publications coded to demand-side or supply-side themes, with 67 publications coded to both (Table 1). Derived from these coded application themes (field 4), Figure 5 presents a structural summary of this bidirectional reconstruction. Specifically, demand-side codes (tourist information search, itinerary generation, and decision support) and supply-side codes (marketing content production, guest-service management, and back-office functions) were aggregated and synthesized into an integrative conceptualization. Functioning to identify mechanisms and contingencies rather than as a causal model estimating causal paths, tracing this evolutionary trajectory of information consumption and production to dissect the mechanisms of new technologies at specific service nodes constitutes the logical starting point for subsequent discussions on commercial efficacy and potential risks.

**Figure 5:**
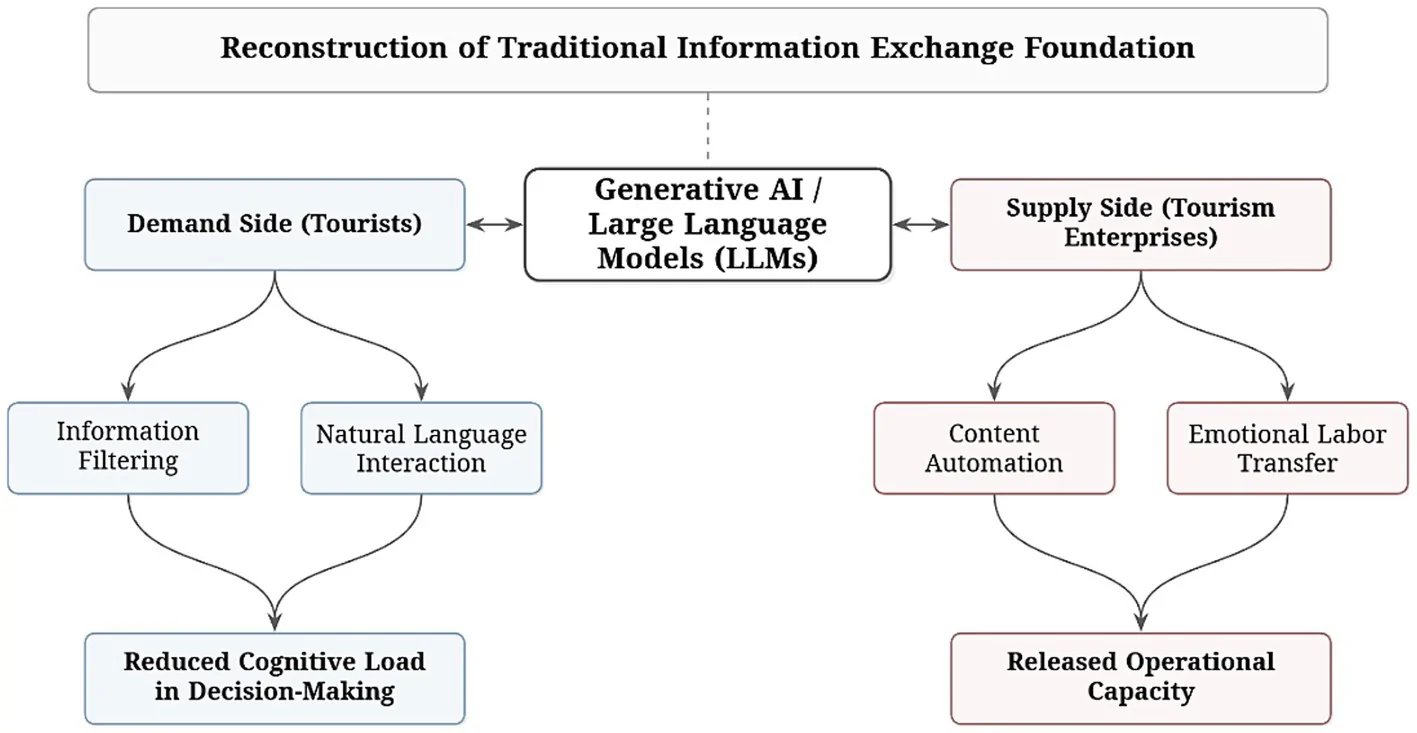
Conceptual framework of the bidirectional reconstruction driven by GenAI in the tourism industry. The figure is an integrative conceptualization synthesized from the coded application themes (*n* = 427 publications coded to demand-side or supply-side applications; Table 1), not a causal model.

**Table 1:** Evidence base for each thematic section.

Section	Theme	Studies (*n*)	Primary designs (*n*)	Main settings	Consistency	Minority findings
4.1	Demand-side information search and itinerary planning	265	Quantitative 171; mixed 40; theoretical/review 32; qualitative 22	Theoretical/unspecified dominant; Global 40; China 12	Consistent	A small minority report accuracy and specificity limits, contingency on user familiarity, and privacy or credibility concerns
4.2	Supply-side operational efficiency and content production	229	Quantitative 113; theoretical/review 46; mixed 45; qualitative 25	Theoretical/unspecified dominant; Global 38; China 22	Consistent	A minority report reduced perceived authenticity of AI-generated content and the need for human correction of AI outputs
5.1	Cognitive and decision-making biases	112	Quantitative 56; mixed 20; qualitative 19; theoretical/review 17	Theoretical 45; Global 22	Consistent	A minority find error concerns do not suppress use in low-risk contexts, and a few note bias-mitigation potential of AI content
5.2	Privacy trade-offs and security	83	Quantitative 43; theoretical/review 19; mixed 12; qualitative 9	Theoretical 27; Global 19	Consistent	A minority show that transparency or source disclosure can backfire by increasing algorithm aversion, and that privacy concerns may attenuate after adoption
5.3	Practitioner dilemmas and organizational adaptation	73	Theoretical/review 26; quantitative 25; mixed 12; qualitative 10	Theoretical 30; Global 9; United States 5	Largely consistent	A minority foreground accuracy, cost, or digital-exclusion concerns rather than ethical risks
6	Authenticity and emotional value	201	Quantitative 105; mixed 45; qualitative 28; theoretical/review 23	Theoretical 68; unspecified 48; Global 24; China 19	Mixed	Two minority streams diverge: studies reporting unqualified positive emotional effects of AI anthropomorphism, and studies reporting direct authenticity loss

### 4.1 Tourist information search and itinerary planning from a demand-side perspective

The synthesis in this section draws on 265 publications coded to the demand-side theme, predominantly quantitative (171) and mixed-method (40) studies with a large theoretical or region-unspecified share (Table 1). Drawing on this corpus, we observe that the intangibility of tourism products compels tourists to conduct intensive pre-trip information searches to hedge against decision-making risks. Constrained by keyword matching logic, traditional online travel platforms and search engines require users to deconstruct systematic travel intentions into fragmented search tags (Kumamoto and Joho, 2025; Xiao et al., 2026). Formulating these search queries forces users to essentially bear the costs of intent translation and the pressure of information filtering (Wong et al., 2023). Tourists must repeatedly cross-verify information nodes across multiple webpages. This passive information acquisition mode easily induces information overload, elevating the cognitive depletion associated with pre-trip decisions (Deng et al., 2026).

Iterations in interactive interfaces alter information search behaviors. Transcending traditional static information indexing, GenAI intervenes in itinerary planning as a dynamic intermediary equipped with contextual comprehension capabilities (Chen et al., 2025). Conversational scenarios fundamentally reshape human-computer adaptation. Using everyday language within a single dialogue box, tourists can articulate compound needs encompassing budget constraints, specific preferences, and time schedules (Shin et al., 2025; Kim et al., 2026), needs that are often negotiated within households, where family technology-use patterns shape members’ travel intentions (Rashidin et al., 2025). Upon receiving unstructured commands, GenAI models parse true intentions via semantic alignment mechanisms, maintaining contextual memory across multi-turn dialogues to execute dynamic corrections (Fang et al., 2025). The algorithm autonomously extracts and integrates dispersed network knowledge. By restructuring fragmented content that previously required manual filtering into structured proposals, it reduces information verification and comparison costs during the pre-trip stage (Nikas et al., 2025).

Beyond text processing, the superimposed generation of multimodal content alters information transmission efficacy. When pure text guides incorporate visual images, geographic coordinates, and dynamic interactive feedback, tourists’ cognitive barriers to unknown destinations correspondingly decrease (Wang and Yoon, 2025; Lu et al., 2026). Multi-dimensional information presentation enhances the utility of itinerary planning while simultaneously amplifying users’ immersive perception of the recommended proposals (Egger and Yu, 2026). As tedious data filtering and content splicing tasks transfer to AI agents, tourists are liberated from the pressure of processing massive information volumes. This offloading of basic cognitive labor allows individuals to focus their mental bandwidth on the in-depth evaluation of recommendations (Park et al., 2025). Their participatory focus shifts from information gatherers to decision-makers evaluating generated proposals.

However, leaps in information processing efficiency do not entirely eliminate decision-making hurdles. Although GenAI shows broad acceptance during broad explorations for travel inspiration, recommendation outputs face rigorous scrutiny when high-involvement decisions—such as specific bookings—are involved (Kim et al., 2025; Bingöl, 2026; Dong et al., 2026; Won and Lee, 2026). Confronting the homogenized suggestions frequently generated by the system, tourists occasionally fall into new choice overload dilemmas when attempting to narrow their options (Mladenović et al., 2024). Consequently, individual digital literacy, algorithmic trust, and pre-existing knowledge of the destination collectively constrain the ultimate efficacy of human-AI interaction models (Fakfare et al., 2025a; Halibas et al., 2026; Zhao et al., 2026).

### 4.2 Operational efficiency optimization and content production from a supply-side perspective

This section synthesizes 229 publications coded to the supply-side theme, dominated by quantitative (113), theoretical/review (46), and mixed-method (45) studies (Table 1). Drawing from this literature, we observe that shifts in demand-side behavioral patterns are accompanied by a synchronous operational restructuring on the supply side. Facing consumers’ expectations for 24/7 real-time interaction and customization, traditional service response mechanisms relying on intensive human labor face critical bottlenecks. To break through these capacity constraints, tourism enterprises and destination management organizations (DMOs) are progressively incorporating GenAI as a critical infrastructure underpinning their foundational operations (Almeida and Ivanov, 2024; Li et al., 2025). Leveraging deep semantic parsing and content generation capabilities, GenAI models are taking over portions of knowledge-intensive and emotion-intensive labor, thereby triggering a reconfiguration of the service value chain (Yaşar and Yayla, 2025). This study summarizes the core application scenarios and efficacy outputs on the supply side in Table 2.

**Table 2:** Application scenarios and efficacy characteristics of GenAI on the tourism supply side.

Operational dimension	Core application scenarios	Algorithmic mechanism	Efficacy characteristics
Front-end guest services management	Processing online reviews and instant inquiries	Parses historical feedback corpora, anchors sentiment polarity, and generates context-aware text	Transfers frontline employees’ emotional labor and reduces cognitive load (Água et al., 2025; Jeong and Lee, 2024; Xu et al., 2026; Önder, 2026)
Digital marketing and promotion	Producing cross-lingual copywriting and multimodal promotional materials	Integrates cross-lingual semantic alignment with text-to-image generation technologies	Shortens production cycles, supports high-frequency marketing tests, and achieves hyper-personalization (Chen and Zhou, 2026; Seo et al., 2025; Florido-Benítez, 2024)
Back-office human resources	Knowledge management and internal onboarding training	Extracts organizational operational data to automatically generate standard operating procedures (SOPs) and dialogue simulations	Fills organizational efficacy gaps and expands the buffer space for human resource allocation (Stergiou, 2025; Bilgihan et al., 2024)

Evidence counts for each row are reported in Table 1; article-level details are provided in the supplementary repository.

In the micro-interactions of customer relationship management, algorithmic systems autonomously generate standardized texts that align with the brand voice, providing customized responses to specific facility or equipment failures. The transfer of emotional labor to AI agents alleviates the cognitive load on frontline practitioners, allowing their energy to flow back into handling complex, physical offline customer complaints (Xu et al., 2026). Cross-lingual generation capabilities dissolve the cultural dissemination barriers faced by DMOs, while multimodal technologies lower the production costs of visual promotional materials. An algorithm-driven content matrix empowers enterprises to conduct high-frequency marketing tests, pushing destination marketing from an experience-led approach into a data-synergized phase of hyper-personalization (Florido-Benítez, 2024; Zhang et al., 2024).

The upgrading of front-end services necessitates a corresponding alignment within the internal management architecture. Addressing the industry’s structural dilemma of labor shortages, enterprises introduce generative tools to optimize knowledge accumulation and training workflows—such as automatically drafting standard operating procedures (SOPs) or constructing simulated complaint scenarios to assist new employee training (Stergiou, 2025; Gedecho et al., 2027). The digital expansion of managerial authority alters established work paradigms. From the perspective of Conservation of Resources theory, when online travel agency employees weigh the benefits of technological assistance against potential substitution risks, the anticipated loss of work resources easily induces deep-seated job insecurity (Lu et al., 2024; Selem et al., 2027; Wei et al., 2026). This psychological anxiety at the cognitive level substantively translates into turnover intentions (Seyfi et al., 2026). Organizational support assumes a buffering role in navigating this technological adaptability crisis, testing the enterprise’s governance capacity to reshape the boundaries of human-AI collaboration (Wang and Zhang, 2025).

While automated tools propel content production efficiency, this singular, efficiency-driven operational logic stands in tension with the tourism industry’s demand for personalized experiences. An overreliance on algorithmically batch-generated text manifests in semantic homogenization; prolonged interaction can easily dilute a distinct brand voice (Giri et al., 2026). Furthermore, when confronting overly perfect, flawless standardized content, consumers possessing high digital literacy are prone to experiencing the psychological phenomenon known as the uncanny valley effect (Loureiro et al., 2025). The experiential distortion associated with the deep intervention of non-human agents challenges the interpersonal core of hospitality upon which the tourism industry depends. Consequently, maximizing operational capacity while preserving interpersonal authenticity during service interactions constitutes a core managerial challenge in advancing intelligence strategies (Saxena and Rishi, 2025).

## 5 Ethical risks and organizational management dilemmas derived from technological application

While the introduction of GenAI into the tourism industry enhances information processing efficiency, the underlying models are probabilistic systems that carry inherent uncertainty and bias, and their flaws propagate into service scenarios as ethical and managerial dilemmas. To organize this terrain, we adopt a three-tier ethical classification grounded in the evidence coverage of the reviewed sample. The first tier comprises risk dimensions that are well documented in the corpus and are synthesized in depth below: cognitive and decision-making biases (112 publications), privacy and security concerns (83 publications), and practitioner and organizational dilemmas (73 publications). The second tier comprises dimensions with growing but thinner evidence in the tourism-specific corpus, which are summarized rather than synthesized in depth: legal responsibility and accountability (28), destination misinformation and fabricated content (20), vendor dependence (19), environmental costs of model training and inference (5), accessibility and digital divides (5), cybersecurity (1), and prompt manipulation (1). The third tier comprises concerns raised in the broader AI literature but not yet evidenced in this corpus—copyright ownership, fabricated reviews, deepfakes, cultural appropriation, employee surveillance, and the concentration of technological power (0 publications each)—which we therefore treat as a research agenda rather than as synthesized findings. This classification makes the inclusion and exclusion of dimensions explicit: a dimension is synthesized only when the corpus provides substantive evidence, and dimensions without corpus evidence are reported transparently as gaps. The same risks affect different stakeholders asymmetrically. Tourists bear misinformation, privacy loss, and eroded trust; employees bear job insecurity, skill obsolescence, and surveillance; firms bear compliance burdens, vendor dependence, and liability; destinations and local communities bear popularity-driven spatial concentration, cultural misrepresentation, and environmental loads; and regulators confront accountability and disclosure questions. Table 3 organizes the first-tier dimensions by risk mechanism and consequence, and the second- and third-tier dimensions are catalogued in Table 4.

**Table 3:** Ethical risks and management dilemmas derived from GenAI technologies.

Risk dimension	Core manifestations	Triggering mechanisms	Industrial and organizational consequences
Cognitive and decision-making biases	AI hallucination and popularity bias	Underlying probabilistic models fabricate non-factual content; training corpora over-rely on historical trending data	Damages tourist trust; exacerbates the over-concentration of spatial tourist flows, hindering the development of marginalized communities (Kim et al., 2024; Zhang and Tong, 2026)
Privacy trade-offs and security	Excessive data extraction and algorithmic black boxes	Personalized services rely heavily on fine-grained preferences; decision-making logic lacks transparency and explainability	Induces technology threat avoidance and defensive psychology; increases enterprise risks regarding data compliance and ethical accountability (Chakraborty et al., 2025; Huang et al., 2025)
Organizational adaptation and skills	Job insecurity and skill obsolescence	Algorithms assume basic informational and emotional labor; internal delineation of rights and responsibilities remains ambiguous	Ruptures labor-management psychological contracts, inducing turnover intentions; weakens the professional capacity of frontline employees to handle complex customer complaints (Seyfi et al., 2026; Zhang et al., 2026)

Evidence counts: cognitive and decision-making biases, *n* = 112; privacy trade-offs and security, *n* = 83; organizational adaptation and skills, *n* = 73. Second- and third-tier dimensions are catalogued in Table 4.

**Table 4:** Emerging and unobserved ethical dimensions of GenAI in tourism and hospitality.

Dimension	Tier	Publications (*n*)	Affected stakeholders
Legal responsibility and accountability	2	28	Firms, employees, regulators
Destination misinformation and fabricated content	2	20	Tourists, destinations
Vendor dependence	2	19	Firms
Environmental costs	2	5	Destinations, communities
Accessibility and digital divides	2	5	Tourists, communities
Cybersecurity	2	1	Tourists, firms
Prompt manipulation	2	1	Firms, tourists
Copyright ownership	3	0	Firms, creators
Fabricated reviews	3	0	Tourists, platforms
Deepfakes	3	0	Tourists, destinations
Cultural appropriation	3	0	Communities, destinations
Employee surveillance	3	0	Employees
Concentration of technological power	3	0	Firms, regulators, communities

### 5.1 Cognitive and decision-making biases induced by algorithmic information distortion

The evidence reviewed in this section comprises 112 publications, mainly quantitative (56) and mixed-method (20) studies. The dominant finding—that hallucination and algorithmic bias distort destination visibility and weaken tourist trust—is consistent across designs, with a minority of studies reporting that error concerns do not suppress use in low-risk contexts (Table 1). Grounded in this literature, we observe that the intangibility of tourism products dictates that tourists rely on accurate information to hedge against unknown risks. Grounded in probabilistic distributions, the text-generation mechanisms of GenAI models introduce new uncertainties into the information acquisition process. While outputting coherent text, GenAI is prone to fabricating non-factual content—a phenomenon referred to in the literature as “AI hallucination” (Christensen et al., 2025). When tourists apply false or outdated information to their itinerary planning, a discrepancy arises between their psychological expectations and the actual environment (Ayyildiz et al., 2025; Belanche et al., 2026; Kim and Lee, 2026). The direct consequence of this expectation gap manifests as a significant decline in users’ trust and perceived usefulness of the technological system (Kim et al., 2024). Cognitive dissonance triggered by this information distortion does more than interfere with individual decision-making; it prompts users to develop defensive psychological mechanisms toward the platform, thereby degrading overall service evaluations (Kumar and Malhotra, 2025). Particularly for tourists lacking background knowledge of a destination, blindly following system recommendations entails bearing higher travel risks and sunk costs (Bui et al., 2025).

Once amplified by algorithmic recommendation systems, cognitive biases at the individual level objectively disrupt the spatial distribution of tourist flows. Because generative models are trained on existing web texts, algorithms naturally assign disproportionate weight to traditional landmarks and popular tourism hubs when processing recommendation requests (Mellors, 2026). Driven by this popularity bias, the system outputs highly homogenized itinerary suggestions (Paül i Agustí, 2025). A parallel logic operates within image generation; destination visual content constructed by GenAI frequently replicates specific cultural stereotypes, failing to authentically capture local diversity (Zhu et al., 2025; Chen et al., 2026). As masses of tourists base their travel decisions on highly redundant information, foot traffic becomes hyper-concentrated in a select few scenic areas. This over-concentrated traffic increases the environmental burden on popular destinations and simultaneously makes it difficult for emerging or marginalized communities to capture corresponding tourism revenues, interfering with the strategic goal of spatially balanced development in the tourism industry (Zhang and Tong, 2026; Andreev et al., 2025; Papathanassis, 2026; Luong et al., 2026).

Data biases originating from algorithmic logic create inequalities in cultural representation and resource allocation. The training corpora of mainstream GenAI models are heavily concentrated in specific regions and language families, causing systems to carry cultural and demographic biases when processing cross-cultural information (Manor and Segev, 2025). Driven by these biases, systems describing destination images often attribute positive characteristics to developed regions while attaching prejudiced labels to developing countries or ethnic minority communities (Le, 2026; Lu et al., 2026). Such distorted objective destination images obstruct authentic communication between different cultures. On the industrial supply side, developing and fine-tuning underlying GenAI models demands substantial computational and capital investments, implicitly raising the barrier to technological adoption for small tourism enterprises or minority operators (George and Mattathil, 2025). Disparities in resource acquisition capabilities widen the digital divide among enterprises of different sizes and restrict the ability of marginalized communities to leverage new technologies for cultural dissemination, compelling academia and industry to embed social equity as a core consideration during the algorithmic deployment phase (Jaiswal et al., 2026; Safari, 2026). Frugal AI pathways—leveraging existing foundation models and lightweight fine-tuning rather than in-house model development—offer resource-constrained hospitality organizations a means of narrowing this adoption divide (Rashidin et al., 2026).

### 5.2 Privacy trade-offs and security considerations in a data-driven context

This section synthesizes 83 publications, predominantly quantitative (43) with a sizable theoretical share (19); the dominant pattern is that privacy concerns, algorithmic opacity, and perceived risk weaken trust and adoption intentions, while a minority of studies show that transparency or disclosure can itself increase algorithm aversion (Table 1). Consistent with this overarching pattern, we observe that achieving precise itinerary planning via GenAI relies on mining massive volumes of tourist behavioral data alongside fine-grained preferences. Because the system’s output personalization is positively correlated with the breadth of user input, this underlying operational logic cements a structural contradiction between customized services and personal information protection (Morosan, 2026). Within tourism service scenarios, securing highly matched recommendations requires tourists to actively embed sensitive information—such as budget constraints, unique dietary habits, and even the demographic characteristics of accompanying family members—directly into their prompts (Aransyah et al., 2026). Because personal data constitutes a psychological and physical resource requiring protection, tourists essentially accept an implicit privacy trade-off when extracting search conveniences from GenAI (Domínguez and Rodríguez, 2025; Jeong et al., 2026). Extracting excessive user data in pursuit of hyper-personalization has been linked to mechanisms outlined in Technology Threat Avoidance Theory, prompting tourists to develop defensive psychological barriers during initial adoption (Chakraborty et al., 2025). Although robust privacy protection designs can positively influence users’ perceived trust, the boundaries and rationale for data collection remain absolute prerequisites determining whether these systems achieve widespread adoption (Abou-Shouk et al., 2024).

The anthropomorphic traits of natural language interfaces effectively lower communication barriers while simultaneously blurring the boundaries between conventional information retrieval and private conversation, leading tourists to inadvertently over-share private details. As interactions deepen, the latent risks of data security and privacy breaches gradually surface, transforming into core variables that inhibit technological adoption. Grounded in the Extended Parallel Process Model, research indicates that when confronting opaque systems like GenAI, privacy and security concerns amplify tourists’ threat perceptions, thereby inducing maladaptive behaviors such as usage avoidance (Yhee et al., 2025). Within specific tourism planning contexts, perceived privacy risk extends beyond merely degrading trust in recommendations; it acts as a negative moderator, actively obstructing the conversion path between social presence and tourists’ continuance intention (Fakfare et al., 2025b; Wang, 2025; Tay et al., 2026). The cognitive and emotional barriers triggered by data security anxieties shape users’ loyalty toward GenAI and their ultimate behavioral intentions (de la Cruz et al., 2026).

Security considerations inherent in this data-driven model extend to the industrial supply side, forming a formidable barrier for tourism enterprises attempting to advance intelligence strategies. When planning GenAI integration, hotels and DMOs are constrained not only by the capital costs of initial deployment but also by severe latent data compliance risks. Lacking clear algorithmic regulations and data protection guidelines, practitioners maintain a highly cautious stance regarding the legality of leveraging consumer data to train domain-specific large models (de la Cruz et al., 2026). Pervasive industry concerns surrounding privacy breaches and ethical accountability severely dampen corporate willingness to leverage these new technologies for optimizing customer service and marketing operations (Huang et al., 2025). In back-office operations such as human resource recruitment, ensuring that systems adhere to data protection and transparency principles when processing candidates’ sensitive information tests the legitimacy of organizational governance (Kwok et al., 2025).

The inherent opacity of algorithmic models during information processing constitutes another critical mechanism driving public technological anxiety. Once tourists input sensitive data, they remain blind to how the system assigns weights to various features to ultimately generate recommendations; this void in explainability severely limits users’ sense of control. Psychological anxiety stemming from this lack of algorithmic transparency actively dilutes the positive utility of GenAI in stimulating travel inspiration (Abou-Shouk et al., 2024). To rebuild the foundational trust in human-AI interactions, scholars propose introducing controllability and transparency features during the algorithmic deployment phase, empowering users to intervene in or trace the generative logic behind recommendations (Fang et al., 2025; Roy et al., 2026). Elevating system explainability serves as an effective mechanism for alleviating user data anxiety. Furthermore, it constitutes a necessary precondition for the tourism industry to transition GenAI from an opaque digital intermediary into a reliable decision-making tool (Ayyildiz et al., 2025; Duong et al., 2025; Shen and Yu, 2025; Korankye et al., 2026; Bu et al., 2027).

### 5.3 Practitioner dilemmas and organizational adaptability under the impact of automation

The evidence base for this section comprises 73 publications, led by theoretical/review (26) and quantitative (25) contributions. The dominant finding on job insecurity, skill reshaping, and governance dilemmas is largely consistent, with a minority foregrounding accuracy, cost, or digital-exclusion concerns (Table 1). Specifically, while elevating operational automation, GenAI simultaneously redefines traditional employment relationships and workflows within the tourism industry. The substitution of repetitive administrative tasks and basic guest inquiries alters employees’ expectations regarding their career trajectories. Facing GenAI interventions, employees at online travel agencies re-evaluate the stability of their current positions (Zhao et al., 2024; Shen et al., 2026b). When frontline staff perceive the latent substitution risks of technology as higher than its auxiliary benefits, deep-seated job insecurity is easily triggered (Carvalho and Ivanov, 2024). This anxiety extends beyond psychological discomfort, actively translating into turnover intentions and signaling that technological deployment is dismantling traditional labor-management psychological contracts (Wang and Zhang, 2025). Although organizational support mitigates employees’ technological anxiety to some extent, it fails to fully counteract the skill panic stemming from reconfigured job responsibilities (Bilgihan et al., 2024).

The imbalance of skill structures and the subsequent risk of erosion constitute another core challenge for practitioners following technological intervention. Because GenAI assumes a vast amount of entry-level information processing, practitioners easily develop an overreliance on algorithmically generated standardized proposals, causing their actual capability to handle complex, non-standardized customer complaints to decline (Almeida and Ivanov, 2024). Engaging in prolonged cognitive offloading can weaken employees’ critical thinking and decisional sensitivity, with the risk of inducing skill obsolescence (Zhang et al., 2026; Selem et al., 2027; Wei et al., 2026). During service recovery phases that heavily depend on interpersonal empathy, algorithmic templates ensure rapid response times but simultaneously restrict the space for individual creativity, structurally damaging the professional service thresholds crucial to the industry’s survival (Xu et al., 2026). Such skill degradation lowers individual market competitiveness and injects latent structural risks for enterprises when confronting future crises or unconventional emergencies.

As human-AI collaboration transitions into a permanent norm, the ambiguity surrounding accountability within service delivery increasingly surfaces, posing a severe test for internal institutional frameworks. When automated replies or intelligent recommendations trigger customer disputes, clarifying whether liability rests with the underlying algorithm providers, frontline operators, or organizational management remains administratively undefined in both academia and industry (Yaşar and Yayla, 2025; Ozdemir, 2026; Mladenović et al., 2026b). This misalignment between authority and responsibility is associated with decisional hesitation among frontline staff and with avoidance behaviors rooted in anxieties over bearing the consequences of algorithmic errors (Zhao et al., 2024). To bridge these institutional voids, some organizations attempt to integrate international ethical frameworks, such as the OECD AI Principles, to guide daily operations; however, actual implementation remains constrained by the dual dilemma of insufficient transparency and ambiguous compliance pathways (Kwok et al., 2025).

While pushing forward intelligent transformations, organizations must synchronously guard against the risks of managerial failure induced by technological black boxes. When GenAI is applied to recruitment screening, performance appraisals, or standard operating procedure generation, the opacity of its decision-making logic exacerbates latent biases within managerial decisions (Stergiou, 2025). This lack of algorithmic transparency amplifies employee skepticism regarding the fairness of internal governance, actively eroding the organization’s foundational trust (Seyfi et al., 2026). To maintain the legitimacy of operational mechanisms, enterprises urgently need to construct explainable, Responsible AI frameworks, ensuring that technological adoption does not occur at the expense of social equity and employee wellbeing (Jaiswal et al., 2026; Zeqiri and Youssef, 2026; Zaidi et al., 2026). Enhancing system explainability and incorporating participatory management mechanisms have become essential pathways for the tourism industry to achieve technological adaptability and transition toward sustainable organizational development.

## 6 Conflicts between authenticity and emotional value in service interactions

The application scope of GenAI in the tourism industry has expanded from basic information retrieval to front-end service interactions. Operating fundamentally as an experience economy, tourism and hospitality services rely heavily on interpersonal interactions to deliver emotional care. As GenAI models see widespread deployment—assuming communication roles traditionally dominated by humans—machine intervention encroaches on the core “human-centric” attributes championed by the tourism industry. Investigating the interplay between technological agents and interpersonal emotions—and dissecting how machine anthropomorphism reshapes tourists’ emotional experiences—constitutes the theoretical premise for answering the third core research question (RQ3). The findings synthesized in this section rest on 201 publications whose coded content addressed authenticity and human touch (field 6, Table 1). Derived from these codes, Figure 6 presents the process model of the interactional layer. Specifically, the coded findings were grouped into parasocial interaction, expectancy violation, algorithm aversion, and human-in-the-loop mechanisms, which constitute the stages of the evolutionary path shown in the figure. Serving as an integrative conceptualization synthesized from the evidence rather than a causal model, Figure 6 identifies mechanisms and contingencies but does not claim to estimate causal paths.

**Figure 6:**
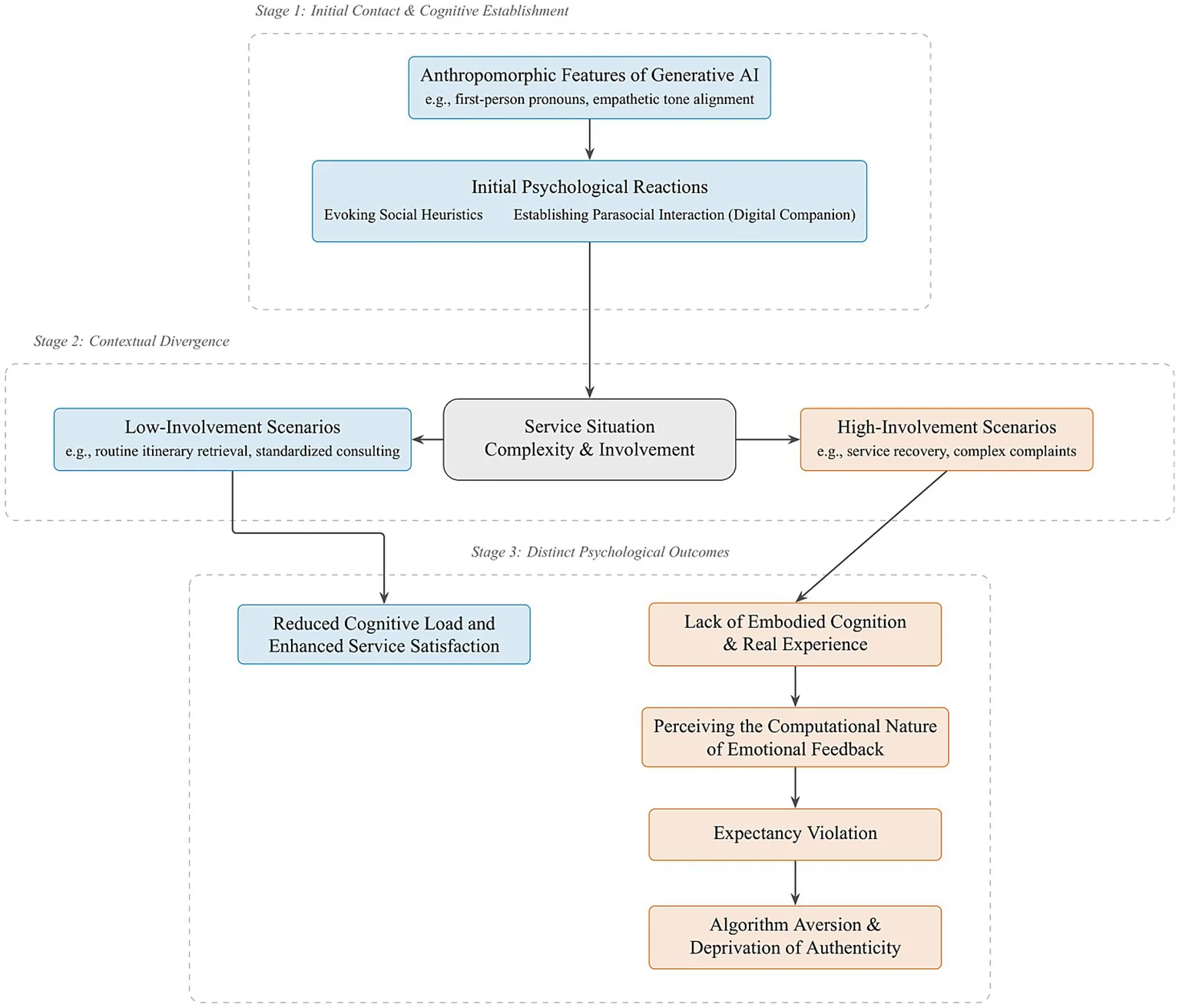
The evolutionary path of emotional interactions between tourists and GenAI. The figure is an integrative process model synthesized from the authenticity and human-touch codes (n = 201 publications; Table 1), not a causal model.

Authenticity in tourism research is a contested construct, and its uses in the reviewed literature vary. Objective authenticity concerns the genuineness of toured objects and places; constructive authenticity treats authenticity as projected and negotiated rather than given (Wang, 1999); existential authenticity refers to the authentic self-states activated by tourism activities; brand authenticity concerns whether a brand’s actions are perceived as consistent, sincere, and true to its identity; and interactional authenticity, the dimension most exposed to algorithmic intervention, concerns the perceived genuineness of interpersonal exchange in service encounters. In this review, the terms authenticity and service authenticity refer to interactional and brand authenticity unless stated otherwise, because the conflict examined in Section 6 arises at the level of service interaction rather than that of toured objects.

### 6.1 Parasocial interaction and the establishment mechanisms of technological emotional value

Emotional communication in tourism services traditionally relies on the authentic empathy of service personnel; however, the evolution of natural language processing technology has endowed algorithmic systems with the computational capacity to simulate human communication styles. By frequently utilizing first-person pronouns paired with emotional alignment strategies during interactions, GenAI models exhibit pronounced anthropomorphism to tourists. Upon receiving highly anthropomorphic conversational cues, the human brain spontaneously activates social heuristics (Jia et al., 2026). Grounded in Parasocial Interaction Theory, tourists confronting digital agents with empathetic tones will unconsciously invoke psychological scripts from daily interpersonal exchanges to forge a unidirectional emotional bond with the AI system (Morosan, 2026; Loureiro et al., 2025; Rahman and Desai, 2026). Within itinerary planning scenarios, the system identifies tourists’ anxieties or anticipatory emotions and outputs reassuring text, which can provide a form of emotional support (Nikas et al., 2025). Tourists on the receiving end of this emotional feedback shift their cognitive positioning, reframing what originated as a mere search tool into a Digital Companion imbued with social attributes (Loureiro et al., 2025).

However, the efficacy of AI agents in providing emotional support is constrained by the complexity of the interactive context. During low-involvement routine itinerary arrangements, algorithmically simulated empathetic responses can effectively reduce tourists’ cognitive load and elevate service satisfaction (Kim et al., 2024). If the interaction scenario transitions into a high-stakes service recovery phase, algorithmic models lacking real-life experience and Embodied Cognition struggle to properly navigate complex emotional conflicts (Wu et al., 2026). System-generated words of comfort expose a programmatic, mechanical nature under such high-pressure scenarios. When tourists seeking authentic emotional resonance detect the computational essence driving this feedback, they experience an Expectancy Violation, triggering the deconstruction of the previously established parasocial interaction (Yaşar and Yayla, 2025). Accompanied by the erosion of foundational trust, algorithmically simulated empathy fails to provide solace; instead, it provokes psychological resistance and aversion among tourists (Loureiro et al., 2025).

This deconstruction of the interactive relationship indicates that digital systems’ simulation of emotional feedback remains constrained by pre-programmed logic and cannot serve as an equivalent substitute for interpersonal emotional expression rooted in genuine human experience. Clearly delineating the applicable scope for algorithms to provide emotional support becomes a prerequisite for the technology to create incremental value without destroying service authenticity (Água et al., 2025; Florido-Benítez, 2024). To achieve this, tourism enterprises deploying GenAI urgently need to establish buffering mechanisms between the system’s highly efficient responses and the authentic touch of physical services; employing granular scenario segmentation to prevent algorithmic over-intervention from diluting the core traits of human-centric service stands as an important managerial priority for sustaining the industry’s core competitiveness.

### 6.2 Theoretical explanations for algorithm aversion and the deprivation of authenticity

Given that the service value of the tourism industry relies heavily on emotional transmission during interpersonal interactions, applying GenAI to customer interaction phases sparks a fundamental conflict between the emotionless computational nature of underlying models and tourists’ psychological expectations for genuine human connection (Yaşar and Yayla, 2025). When reading algorithmically generated text, consumers’ acute perceptual abilities allow them to detect the absence of authentic empathy behind the dialogue (Xu et al., 2026). Because generative content is exceptionally low-cost and highly standardized, tourists perceive it merely as the output of code rather than an exclusive emotional investment tailored to their individual circumstances (Saxena and Rishi, 2025; Zhang et al., 2024; Gu and Spann, 2026). Upon detecting this deficit in emotional labor, tourists correspondingly downgrade their evaluations of service authenticity, forcing AI agents into a trust dilemma when navigating high-emotion demands (Giri et al., 2026).

Disclosing the algorithmic origin of content constitutes the critical mechanism triggering trust decay. When the algorithmic source remains undisclosed, highly simulated text can still provide temporary psychological comfort; however, the moment tourists learn that a letter of care or a service recovery proposal was actually generated by code, they experience an Expectancy Violation (Aransyah et al., 2026). Realizing they are confronting computationally simulated emotions, tourists interpret the enterprise’s service as perfunctory, eliciting Algorithm Aversion (Tan et al., 2025; Shi et al., 2026). Compared to purely instrumental tasks prioritizing efficiency, the core of tourism services lies in emotional resonance, rendering Algorithm Aversion more pronounced in these scenarios (Wang et al., 2026). Algorithmic intervention in emotional maintenance fractures the reciprocity principle of interpersonal relationships, generating psychological resistance toward the brand during subsequent interactions (Ali et al., 2025).

To counteract Algorithm Aversion and authenticity deprivation, introducing Human-in-the-loop mechanisms has become a central avenue for sustaining brand value. While algorithms possess clear advantages in processing massive datasets and drafting text, they cannot bear moral responsibility or deliver Embodied Empathy (Jia et al., 2026; Huang et al., 2026). At critical service nodes such as customer complaint resolution, human employee intervention does more than merely correct algorithmic fallacies; it injects authentic emotional care into the service delivery process. Positioning AI agents as back-office auxiliary tools while tasking frontline employees with ultimate emotional transmission helps preserve the essence of Hospitality crucial to the tourism industry, without sacrificing operational efficiency (Wan, 2024; Almeida and Ivanov, 2024).

## 7 Discussion

Through a systematic literature review, this study has deconstructed the commercial efficacy, ethical risks, and managerial dilemmas inherent in the application of GenAI within the tourism and hospitality industry. Transcending the limitations of singular technology adoption models, this section aims to synthesize and elevate the core findings of the entire manuscript. It investigates the underlying tug-of-war between the surge in operational efficiency and the crisis of service authenticity driven by technological application, ultimately providing a substantive reference framework for future academic exploration and industrial practice.

### 7.1 Synthesis and discussion of core findings

The deployment of GenAI in the tourism industry reconstructs its information retrieval, service interaction, and organizational management workflows. Synthesizing the analyses across various application scenarios indicates that while GenAI models optimize information processing efficiency on both the supply and demand sides, they simultaneously spawn challenges regarding technological ethics and service authenticity (Tuyen et al., 2025). While the technology expands corporate capacity and diminishes tourists’ cognitive burdens, its underlying logic rooted in probabilistic distributions and heavy reliance on massive datasets render cognitive distortion, privacy concessions, and emotional deprivation systemic risks coexisting with technological efficacy (Carvalho and Ivanov, 2024; Buitrago-Esquinas et al., 2024). A balanced evaluation of the utilities and systemic costs brought by this technology establishes a theoretical foundation for understanding the complexities of the current intelligent transformation. To clearly delineate this trade-off, this study outlines the core findings within an efficacy-risk hedging matrix (see Table 5).

**Table 5:** Efficacy-risk hedging matrix of GenAI in the tourism industry.

Application dimension	Core commercial efficacy (benefits)	Systemic derived risks (costs)	Underlying conflict mechanism
Information processing and decision-making	Lowers cognitive depletion; accelerates proposal generation	AI hallucination; popularity bias; cognitive distortion	Mismatch between probabilistic generative logic and precise information demands (Kim et al., 2024; Mellors, 2026)
Service interaction and emotion	Transfers emotional labor; provides preliminary reassurance and support	Expectancy violation; algorithm aversion; authenticity deprivation	Tension arising between computational essence and human-centric empathy demands (Yaşar and Yayla, 2025; Loureiro et al., 2025)
Organizational management and operations	Unlocks operational capacity; optimizes knowledge management	Skill obsolescence; privacy compliance; job insecurity	Tug-of-war between automated expansion and human agency control (Lu et al., 2024; Zhang et al., 2026)

The underlying conflict mechanisms between commercial efficacies and systemic risks across these dimensions are further elaborated in the main text.

During service delivery, a goal divergence persists between algorithmic operational logic and human-centric emotional demands. By batch-generating standardized text and multimodal content, algorithmic agents effectively minimize corporate content production costs and tourists’ information retrieval time (Bilgihan et al., 2024). However, when technological intervention extends into service touchpoints requiring high empathy—such as customer complaint resolution or personalized care—algorithmic models lacking embodied experience struggle to offer authentic emotional feedback (Yaşar and Yayla, 2025). Once service recipients detect the computational essence driving this emotional feedback, the pre-existing foundation of trust faces deconstruction, eliciting the phenomenon of algorithm aversion (Tan et al., 2025). This mechanism indicates that a technological logic singularly pursuing operational efficiency dilutes the service attributes emphasizing authentic interpersonal connections within the tourism industry when handling high-involvement emotional labor (Xu et al., 2026). Failure to meticulously delineate the boundaries of responsibility between AI and human employees in emotional transmission exposes enterprises to the risks of service homogenization and brand equity erosion (Zhao et al., 2024).

The coexistence of efficacy and risk translates into ambivalent and vacillating user attitudes toward technology acceptance. To secure highly precise, customized itinerary recommendations, tourists must input sensitive information—including budgets and personal preferences—into the algorithmic system (Domínguez and Rodríguez, 2025). Confronting opaque black-box algorithms, privacy and security anxieties amplify tourists’ threat perceptions, driving them to adopt defensive behaviors while extracting informational convenience (Yhee et al., 2025; Fakfare et al., 2025b) While the highly anthropomorphic interaction styles of algorithmic agents foster preliminary parasocial interactions, the blurred boundaries between their instrumental and social attributes heighten the instability of user psychological expectations (Chen et al., 2025). As tourists repeatedly weigh efficiency gains against privacy anxieties, and emotional expectations against authenticity deprivation, their adoption behavior toward generative systems manifests a high degree of situational dependency (Volchek and Ivanov, 2024; Xiao et al., 2026). This ambivalent acceptance behavior clarifies why GenAI achieves widespread adoption in pre-trip information searches yet frequently encounters resistance during deep emotional interactions and final decision-making phases (Água et al., 2025).

### 7.2 Theoretical implications

The review carries four theoretical implications. First, it shows that the theories currently invoked in GenAI research are complementary mechanisms of one socio-technical system rather than competing lenses: TAM and UTAUT operate at the utility layer, Conservation of Resources and Technology Threat Avoidance theories at the appraisal layer, and Parasocial Interaction, Expectancy Violation, and embodied-cognition perspectives at the emotional layer of service encounters. Second, the evidence specifies the boundary conditions under which efficiency gains turn into risks: task involvement, emotional intensity, disclosure, digital literacy, and organizational support determine which mechanisms dominate. Third, the authenticity conflict extends authenticity theorizing to algorithmic service contexts, suggesting that interactional authenticity—rather than objective authenticity—is the dimension most exposed to machine intervention. Fourth, the ambivalent acceptance pattern documented across studies calibrates technology acceptance models for generative systems: adoption and aversion are not opposite poles of one continuum but situational responses that can coexist within the same user.

### 7.3 Practical managerial implications: constructing a responsible human-in-the-loop framework

Intelligent transformation reconstructs the service delivery logic of the tourism industry. To navigate the conflict between efficiency expansion and emotional deprivation, tourism enterprises should implement a tiered human-in-the-loop framework in which the degree of automation varies with the stakes of the decision. Routine information retrieval, standardized inquiries, and draft content generation can be automated, with human verification applied to the output; decisions that affect service recovery, personalized care, pricing, or legal exposure require explicit human approval before execution; and escalation is mandatory whenever the system encounters emotional distress, complaints involving liability, or requests outside its trained domain. The framework should also specify authority and accountability: frontline employees hold the authority to reject an AI output, managers hold escalation authority, and the organization—not the algorithm—carries responsibility for errors, so that authority and liability are aligned rather than left ambiguous.

Human involvement is not by itself a guarantee of quality. Employees may accept AI recommendations automatically, lack the expertise to detect errors, or become dependent on generated outputs. The framework therefore embeds supporting safeguards: audit procedures that log AI decisions and human overrides, privacy controls that enforce data minimization and purpose limitation, correction mechanisms that route detected errors back into prompt and workflow redesign, employee training on the system’s failure modes, and continuous monitoring of accuracy, fairness, and escalation rates. The resulting allocation is summarized in Table 6. On the consumer side, managers should clearly label algorithmically generated content on interactive interfaces and grant users actual control over the scope of data access (Yhee et al., 2025; Fakfare et al., 2025b). Disclosure policies, however, are double-edged: labeling may improve transparency while simultaneously increasing algorithm aversion, because disclosed AI authorship lowers perceived authenticity and, in some studies, reduces persuasion (Tan et al., 2025; Carvalho et al., 2025). Disclosure should therefore be combined with demonstrated human oversight rather than treated as a standalone remedy.

**Table 6:** A tiered human-in-the-loop allocation for tourism service delivery.

Decision tier	Automation level	Human role	Safeguards
Routine information retrieval and standardized consultation	High: AI drafts, human spot-check	Approve or correct drafts	Audit log; accuracy monitoring
Personalized recommendations and marketing content	Medium: AI generates, human approves	Approve before release	Disclosure labeling; brand-voice review
Service recovery and high-emotion interactions	Low: AI drafts, human leads	Own the final response and escalation	Escalation protocol; empathy training
Pricing, legal, and strategic decisions	Minimal: AI supports analysis	Full human authority and liability	Compliance review; liability mapping

### 7.4 Reflexive critique of methodological limitations and future exploratory directions

The existing literature relies heavily on cross-sectional questionnaires and static technology acceptance models, remaining disproportionately concentrated in pre-trip, online settings. To address these gaps, we propose five research priorities, each outlining a specific inquiry, theoretical lens, and methodological approach:

1. Disclosure and Trust Mechanisms: Under what conditions does disclosing AI authorship strengthen rather than weaken consumer trust? Grounded in expectancy violation theory and algorithm aversion research, future scenario-based and field experiments should manipulate the timing, framing, and source of disclosure. This individual-level focus within marketing and service recovery contexts will help identify the boundary conditions of transparency, reconciling the contradictory findings documented in Table 5.
2. AI-Generated Service Recovery: How does service failure severity alter consumer reactions to AI-generated recovery messages? By integrating justice theory with expectancy violation theory, experimental designs crossing failure severity with message authorship can clarify when human-led recovery remains indispensable and when AI drafts suffice.
3. Spatial Redistribution of Tourism Demand: Does GenAI steer tourists toward lesser-known destinations or reinforce popularity bias? Utilizing platform-level recommendation logs and quasi-experimental designs, researchers can test whether interface interventions mitigate the concentration effects (Section 5.1), thereby linking algorithmic design directly to destination-level outcomes.
4. Employee Expertise and Cognitive Dependence: How does repeated GenAI use affect employees’ expertise, decision quality, and cognitive dependence over time? Drawing on conservation of resources theory and skill-based views of work, longitudinal field studies in hotel and travel agency settings are needed to trace the long-term accumulation of benefits and the potential erosion of organizational capabilities.
5. Equity and Societal Consequences: The current corpus is notably thin regarding cultural differences, language inequalities, digital literacy, and regulatory environments, and silent on long-term socio-environmental impacts. Future efforts should employ comparative cross-cultural studies, small-firm case studies, and life-cycle assessments of AI infrastructure to connect this review’s framework with broader social sustainability agendas.

Methodologically, future research should move beyond pre-trip digital interfaces: on-trip studies that pair conversational agents with IoT sensors or embodied systems remain scarce (40 publications), and field experiments with behavioral and physiological measures would test whether self-reported scales diverge from actual adoption behavior. Combined with the cross-database tracking strategy recommended in the limitations, these designs would test and extend the socio-technical framework developed in this review.

### 7.5 Limitations of the study

This systematic literature review entails limitations. First, although the search covered the Web of Science Core Collection and Scopus, relevant work in specialized information systems and computer science outlets, conference repositories, and preprint servers may have been missed, and backward and forward citation searching was not performed. Second, the search strings consisted of English terms, and the retrieved records were predominantly in English (1,492 of 1,500 Web of Science records), so findings may not capture patterns reported in other languages or local journals. Third, the static cutoff of September 9, 2026 means that the most recent developments fall outside the sample. Fourth, although a design-sensitive quality appraisal weighted the interpretation of the evidence, 54 publications (11.7%) were appraised as methodologically weak, and some thematic conclusions rest on comparatively small evidence bases (e.g., 73 publications for the organizational theme). Subsequent systematic reviews should combine multilingual search strategies across multiple databases with dynamic tracking mechanisms and backward citation searching to continuously refine and expand the framework established in this review.

## 8 Conclusion

This review synthesized 461 publications on generative artificial intelligence in tourism and hospitality, published between 2021 and September 2026 and identified through the Web of Science Core Collection and Scopus. Its primary contribution is an efficacy-risk hedging framework: application-level benefits on the demand and supply sides, systemic ethical risks, and the authenticity conflict are integrated within one socio-technical account, with each thematic claim anchored in article-level evidence counts and a design-sensitive quality appraisal.

Three principal findings follow. First, GenAI’s value creation is concentrated in information-intensive tasks—information search and itinerary generation, marketing content, and back-office functions—documented by 265 and 229 publications on the demand and supply sides, respectively. Second, these gains are systematically accompanied by risks: hallucination and popularity bias, privacy trade-offs, and practitioner deskilling appear as consistent rather than isolated findings across 112, 83, and 73 publications. Third, in emotionally charged service encounters, machine-generated empathy frequently elicits expectancy violations and algorithm aversion (201 publications), so that the authenticity conflict—rather than efficiency—sets the boundary of GenAI’s value in hospitality. The tiered human-in-the-loop model translates these findings into governance practice.

## Data Availability

The original data presented in the study are openly available in FigShare at: http://doi.org/10.6084/m9.figshare.32494353.
